# Retrospective Radiographic Analysis of Peri-Implant Bone Loss in Mandibular Full-Arch Implant Rehabilitations

**DOI:** 10.3390/diagnostics14212404

**Published:** 2024-10-29

**Authors:** Francesco Giordano, Alfonso Acerra, Roberta Gasparro, Marzio Galdi, Francesco D’Ambrosio, Mario Caggiano

**Affiliations:** 1 Department of Medicine, Surgery and Dentistry, Scuola Medica Salernitana, University of Salerno, Salerno, Via S. Allende, 84081 Baronissi, Italy; frgiordano@unisa.it (F.G.); marzio.galdi@gmail.com (M.G.); macaggiano@unisa.it (M.C.); 2 Department of Neuroscience, Reproductive Sciences and Dentistry, University of Naples Federico II, 80131 Naples, Italy; roberta.gasparro@unina.it

**Keywords:** bone loss around implants, implant-supported prosthetic rehabilitation, mandibular flexure, mandibular full-arch implants

## Abstract

Objectives: Can the type of implant rehabilitation influence peri-implant bone loss in case of full-arch mandibular prosthesis? The purpose of the study was to assess, using orthopantomograms (OPGs), the bone loss around implants in different types of implant-supported prosthetic rehabilitations and identify potential risk factors, associated with the number and location of implants, that may have an association with bone defects. Methods: A radiographic study was conducted on 22,317 OPGs from 2010 to 2024. All OPGs with implant-supported prosthetic mandibular rehabilitations were included in the study. Results: A total of 155 OPGs were evaluated, with peri-implant bone loss identified in 64 (41.3%). Distal implants (furthest from the center) across various positioning patterns were most susceptible to bone loss, with positions 3.6 and 4.6 demonstrating the most frequent occurrence (25 and 26 cases, respectively). The χ^2^ test revealed significant associations between both the implant positioning pattern (*p* < 0.001) and number of implants (*p* < 0.001) with peri-implant bone loss. Also, by updating the sample of OPGs, increased susceptibility to bone resorption was found for implants placed distal to the mental foramen compared to mesial ones in full-arch-implant-supported fixed prostheses. Conclusions: Prospective clinical studies will therefore be useful in investigating this finding further.

## 1. Introduction

With the development of new technologies, edentulous patients have had the opportunity to access implant surgery protocols that include fixed protheses on implants that are positioned in the alveolar bone according to the bone quality and quantity of the patients, the risks of peri-implantitis, and the osseointegration process [1,2,3]. The original protocol of osseointegrated implantology involved implant placement in totally edentulous patients [4,5]. Technological progress has also affected implant surgery, with the development of new methods and techniques leading to computer-guided procedures to program treatments that differentiate according to implant numbers, sites, loading times, and prosthetic structures [6,7,8,9].

Brånemark studied full-arch rehabilitation and stated that the implants in the anterior area of the bone should be parallel to each other, to support a full-arch prosthetic rehabilitation; five implants should be located in the lower jaw, and six should be located in the upper jaw. With this method, the survival rate reached to 90% after 10 years. Then, Branemark et al. compared, in another study, the difference between using four and six implants in full-arch rehabilitation [10]. In this retrospective analysis, 156 patients were recruited and after 10 years, there was no difference in terms of biomechanics. Predictability rehabilitations with a reduced number of implants in cases of totally edentulous patients have been studied, demonstrating that a small number of implants is better in terms of oral hygiene and bone fit because there is a greater distance between them [11,12,13].

In edentulous patients, it is important to state that the bone quality, especially in the posterior sectors, and the anamnesis of the patient are fundamental in cases of systemic disease or smoking habits, because these conditions are often not compatible with implant insertion [14,15,16] (aggiungi articolo lenalidomide). Therefore, in cases of insufficient bone volume, bone augmentation techniques are used. These help to maintain implant stability, improving the biomechanics of the implants in terms of occlusal force distribution with increased predictability of survival and success rate, but this method has disadvantages, such as increased time requirements and cost and, in some cases, biological complications associated with the use of certain types of materials [17,18,19]. The concept of intentionally tilted implants has been proposed as an alternative method [20,21,22]. This procedure can help to reduce cantilevers of the overlying prostheses and achieve a better distribution of implants in the arch [23]. With the aim of reducing the cantilever of distal rehabilitation, this protocol is proposed in cases of completely edentulous posterior sectors in both the upper and lower jaw [24]. Specifically, the desired result is achieved through the use of four implants placed between the two mental foramen at the lower arch and four implants placed between the two anterior walls of the maxillary sinuses at the upper arch. Of these, the two most distal implants are tilted distally to the occlusal plane. With this technique, it is necessary to make implant-supported fixed protheses with distal cantilevers, increasing the stress on peri-implant bone because of the absence of implants distal to the noble anatomical structures [25]. Indeed, peri-implant bone is an area of great bone turnover and an area of great attention for clinicians, as it is most susceptible to the stresses that the implant system undergoes. Any overloads are concentrated specifically on the peri-implant marginal bone [26]. A key factor in the reduced adaptability of the implant system under stress may be the absence of the periodontal ligament [27]. Kim et al., in a review, highlighted that this phenomenon of major remodeling may occur more frequently when occlusal overload persists at the implant level [27]. The mandibular bone, as well as all long bones in the human body, undergo elastic deformation when subjected to functional stress [28]. The particular biology of lower jawbone tissue, its anatomical shape, and its close relationship with the muscle and ligament tissues of the cervicofacial district, specifically the masticatory muscles, represent the main causes of these phenomena [29]. In addition, in edentulous patients, this phenomenon, better known as median mandibular flexure, can influence the implant success rate [30,31,32].

The contraction of the inferior head of the lateral pterygoid muscles is one of the main causes of mandibular deformation, particularly active during opening and protrusive movements [33,34]. Median mandibular flexion (MMF) causes a great reduction in the mandibular arch, and this can lead to peri-implant bone loss in cases of implant rehabilitation [32].

Thus, the choice of the number and placement of implants that will support the patient’s prostheses becomes of paramount importance in implant rehabilitation in cases of totally edentulous arches. Describing the biomechanical difficulties associated with the implant system and the living, continuously remodeling tissue that accommodates this system, the purpose of this study was to assess, through a retrospective radiographic analysis, any correlation between different types of mandibular full-arch implant-supported rehabilitations and peri-implant bone loss. In particular, a comparison was made between rehabilitations that involved implant placement between the mental foramen and those that also involved implant placement distal to it. It is also important to state the possible association between bone resorption and specific risks related to implant number and position to help clinicians to choose the appropriate full-arch mandibular rehabilitation method for each individual case.

The null hypothesis of this study was the absence of significant differences between peri-implant bone loss and the implant placement pattern, number of implants, and implant position.

## 2. Materials and Methods

A retrospective observational analysis was performed on digital panoramic radiographs (OPGs) of 24,330 subjects, taken between January 2010 and April 2024. The OPGs were taken in a private dental clinic based in Salerno, Italy, through Orthophos Sirona (Sirona Dental Systems GmbH, Bensheim, Germany) radiographic device use and analyzed through Sydexis XG 2.1 software (Dentsply Sirona, Charlotte, NC, USA) imaging viewer software. All the images were viewed under the same conditions of light and resolution (27 inches 4k Ultrasharp, Dell Inc, Round Rock, TX, USA). All the files were transferred anonymously.

All the radiographs showed a fixed full-arch-implant-supported mandibular rehabilitation in fully edentulous patients with a minimum follow up of 5 years, up to a maximum of 10 years.

The exclusion criteria were as follows:

- Full-arch rehabilitations with less than 5 years of follow-up;
- Full-arch rehabilitations with more than 10 years of follow-up;
- Mandibular rehabilitations with dental implants supporting removable prostheses;
- Mandibular rehabilitations with dental implants supporting fixed prostheses with horizontal bone resorption;
- Dental elements in the mandible;
- OPGs of low resolution.

Two trained examiners (A.A. and M.G.) with a good level of experience in radiographic evaluation and implantology independently analyzed the OPGs. To guarantee consistency in the evaluation process, the researchers were presented with a randomized assortment of OPGs. The inter-examiner reliability in the data extraction and collection process was assessed using the Cohen kappa coefficient. Inter-observer agreement analysis yielded values exceeding 0.90, indicative of substantial concordance. Intra-observer agreement analysis produced values spanning from 0.87 to 0.99, interpreted as representing a substantial-to-almost-perfect agreement. This study demonstrated robust inter- and intra-observer reliability, signifying high precision in the measurements obtained for both individual evaluators and repeated measurements by the same evaluator.

The sample was divided into 5 patterns, based on the number of implants inserted and the implant location. The division was based on mandibular rehabilitations supported by 4, 6, or 8 implants placed either between, or distal to, the two mental foramina.

In particular,

- Model 1 was characterized by the presence of implants at sites 4.5, 4.2, 3.2, and 3.5;
- Model 2 was distinguished by the presence of implants at sites 4.6, 4,4, 4.2, 3.2, 3.4, and 3.6;
- Model 3 was characterized by the presence of the implants at position 4.6, 4,4, 4.3, 3.3, 3.4, and 3.6;
- Model 4 was distinguished by the presence of implants at position 4.7, 4.6, 4,4, 4.2, 3.2, 3.4, 3.6, and 3.7;
- Model 5 was characterized by the presence of the implants at sites 4.7, 4.6, 4,4, 4.3, 3.3, 3.4, 3.6, and 3.7.

Bone level measurements in the mesial and distal positions were calculated on all OPGs using Sydexis XG 2.1 software (Dentsply Sirona, Charlotte, NC, USA). Following calibration using the known implant diameter, the ruler function was used to measure the distance between the implant shoulder and the bone crest mesial and distal to it. The degree of accuracy was 0.05 mm. In cases of bone loss related to the length of the implant, a peri-implant bone defect was diagnosed. The chosen cut-off was 25% [35].

### Data Collection and Statistical Analysis

Data obtained from the OPGs were reported on an electronic worksheet in Microsoft Excel software 2019 (Microsoft Corporation, Redmond, WA, USA). Frequencies and percentages were calculated for the implant placement model and the number and location of implants. In order to compare frequencies, the χ^2^ test was used to evaluate whether peri-implant bone loss was related to the implant placement model, number of implants, and implant position. Statistical significance was set at a *p*-value < 0.05. Statistical analysis was performed using SPSS software version 28.0 (IBM SPSS, Armonk, NY, USA).

The sample size, calculated using the statistical software G*Power 3.1.9.7, was determined by considering a statistical power of 80% and a significance level α = 0.05 as 63 individuals.

## 3. Results

**Figure 1:**
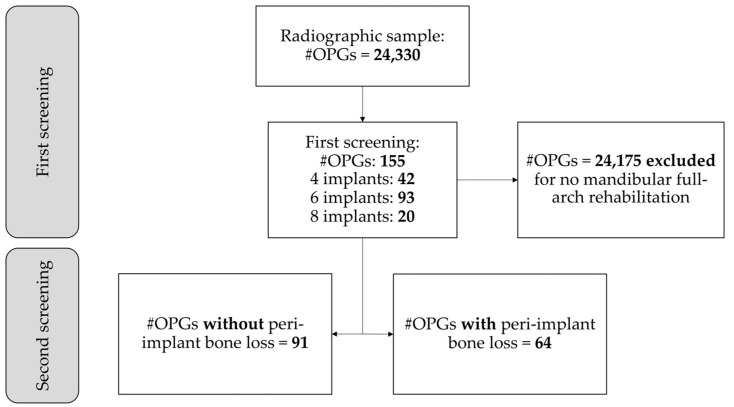
Screening process of OPGs, with a first screening to exclude OPGs without mandibular full-arch rehabilitation and a second screening to assess OPGs in which peri-implant bone loss is present.

The present study included 155 OPGs that met the eligibility criteria, and a peri-implant bone defect was identified in 64 (41.3%) of the 155 OPGs (Figure 1).

The frequencies and percentages of the implants’ position are shown in Table 1.

According to the implant positioning models, 42 (27.1%) full-arch rehabilitations were performed according to pattern 1, 67 (43.2%) followed pattern 2, 26 (16.8%) were positioned according to pattern 3, 13 (8.4%) followed pattern 4, and 7 (4.5%) were placed according to pattern 5.

Peri-implant bone loss was detected in 38 (24.5%) OPGs in which implants were placed with pattern 2, 13 (8.4%) in which implants were positioned following pattern 3, 8 (5.2%) that followed pattern 4, and 5 (3.2%) that followed pattern 5. The type 1 positioning pattern was not associated with peri-implant bone defects.

**Figure 2:**
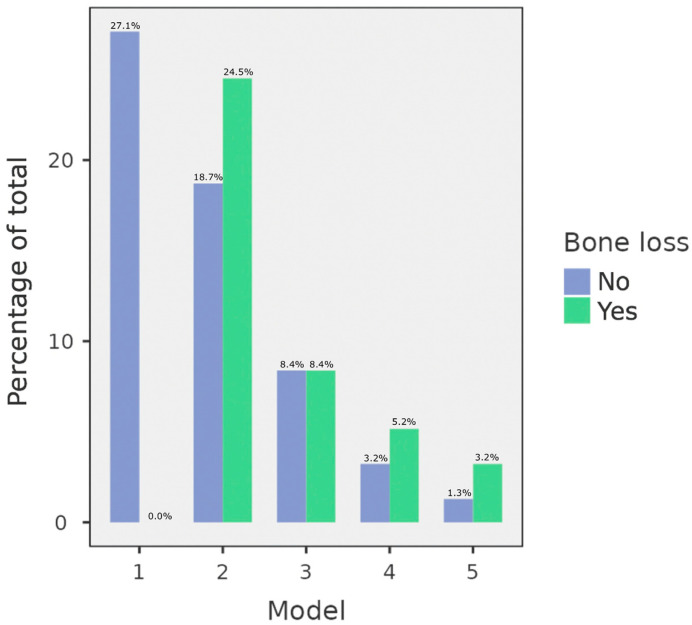
Distribution of OPGs in relation to implant placement patterns and peri-implant bone loss. The blue columns represent those in which no peri-implant bone loss was observed, and the green columns represent those in which it was found.

The χ^2^ test found that there was a statistically significant difference between implant placement pattern and peri-implant bone loss (*p* < 0.001). The distribution of OPGs in relation to implant positioning models and peri-implant bone loss is shown in Figure 2 and Table 2.

Based on the number of implants, the sample consists of 42 (27.1%) full-arch rehabilitations with four dental implants, 93 (60.0%) with six dental implants, 20 (12.9%) with eight dental implants.

No implants presented bone defects in OPGs in which four implants were placed, while 51 (32.9%) OPGs in which full-arch rehabilitation included six implants, and 13 (8.4%) OPGs in which eight implants were placed, exhibited peri-implant bone loss.

**Figure 3:**
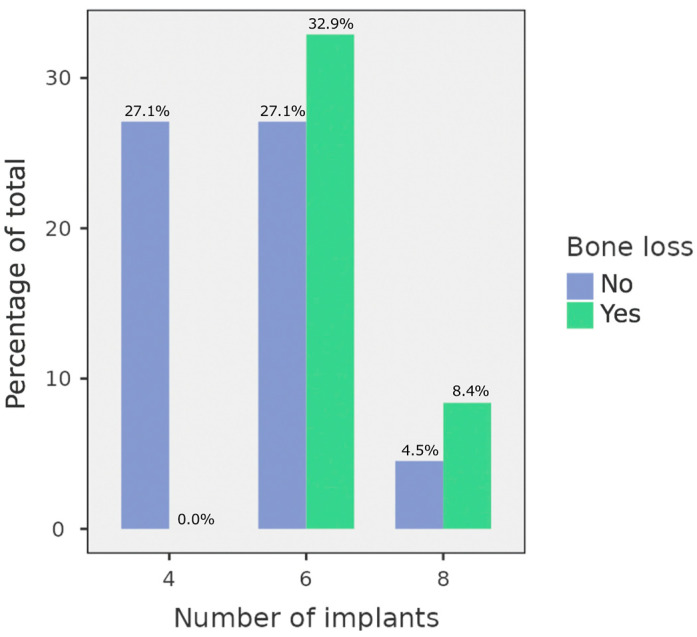
Distribution of OPGs in relation to the number of implants and peri-implant bone defects. The blue columns represent those in which no peri-implant bone loss was found, and the green columns those in which it was observed.

A statistically significant difference between the number of implants and peri-implant bone loss was found (*p* < 0.001) using the χ^2^ test. The distribution of OPGs in relation to the number of implants and peri-implant bone loss is displayed in Figure 3 and Table 3.

**Figure 4:**
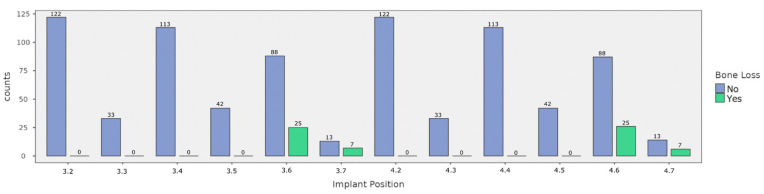
Distribution of OPGs in relation to implant positions and peri-implant bone defects. The blue columns represent those in which no peri-implant bone loss was found, and the green columns represent those in which it was observed.

Peri-implant bone loss was predominantly observed in the models with the number distal implants being 2, 3, 4, and 5. Analyzing implant positions, a total of 64 out of 822 implants exhibited peri-implant bone defects. Of these, 25 (2.8%) were positioned in location 3.6, 7 (0.8%) were positioned in location 3.7, 26 (2.9%) were positioned in location 4.6, and 6 (0.7%) were positioned in location 4.7. In particular, the χ^2^ test revealed a statistically significant difference between implant position and peri-implant bone deficits (*p* < 0.001). The detailed distribution of implant positions according to bone loss is presented in Figure 4 and Table 4.

## 4. Discussion

The aim of this study was to detect peri-implant bone loss in full-arch mandibular fixed rehabilitations supported by different numbers of implants and evaluate potential risk factors associated with the number and location of implants that may have an association with bone defects. In these cases of mandibular rehabilitation, this study shows the prevalence of peri-implant bone loss. In total, 24,330 panoramic radiographs (OPGs) were collected from the initial sample, but not all the OPGs met the inclusion criteria. The rigorous selection process found 155 OPGs for analysis. The resulting sample was therefore found to be representative and relevant to the research goals. A total of 64 digital radiographs showed peri-implant bone loss involving the implant being inserted distal to the mental foramen, an interesting outcome to investigate. In fact, the results of this study showed greater susceptibility to peri-implant bone defects in implants inserted distal to the mental foramen than those mesial to it in cases of full-arch mandibular rehabilitations. These results continue to raise questions about the potential role that the location and number of implants may play in the long-term success of full-arch mandibular rehabilitation [35,36]. Peri-implant bone loss and marginal radio-transparency were identified among the criteria for implant success. In fact, values greater than 1.5 mm during the first 12 months of loading, and 0.2 mm for each year thereafter, indicate the failure of implant rehabilitation [37]. The new 2018 classification of peri-implant diseases and conditions assigns 2 mm as the limit to diagnose peri-implant disease; however, there are different opinions in the literature [38]. This difference in results can be attributed to biomechanical variables, such as loading forces or mandibular flexure, key factors in this area. An association between occlusal overloads and peri-implant crestal bone loss is also described in a systematic review by Di Fiore et al., which, in any case, requires further studies with reproducible and standardized methods to support this relation more strongly [39].

In the patterns that included the placement of at least one implant distal to the mental foramen, there was at least 50 percent bone loss, a finding that was found to be statistically significant.

Mandibular flexion had also been identified by Miyamoto et al. as the main cause of implant failure in mandibular full-arch rehabilitation [40]. In fact, especially during opening and protrusion movements, implants connected together in a rigid rehabilitation capable of resisting mandibular flexion were found to be more susceptible to vestibulo-lingual forces [33,41].

In the crestal area, a stress from the side may affect osseointegration and cause peri-implant bone loss [42,43]. Indeed, it has also been reported that peri-implant bone loss in overload is possible even in the absence of inflammatory phenomena [44]. The absence of the periodontal ligament radically changes the response of the implant system to occlusal overloads, with the lack of an adaptation phase, which is instead present in the periodontal system [45].

Other studies that evaluated the functional loads and deformations caused by mandibular flexure in different types of fixed mandibular rehabilitations also identified the same conclusions. In all described patterns, the more distal implants that supported the prosthetic rehabilitation above were found to be more susceptible to overloads and stresses.

Mijiritsky et al. showed that the area at the level of the crestal module of the distal implants that supports mandibular rehabilitations, mainly in the molar region, has peri-implant bone stress caused by mandibular flexion, as shown by the results of this study [46]. It is important to also consider other factors, in addition to mandibular flexion, that can cause this phenomenon in full-arch rehabilitations of edentulous jaws, to prevent them from negatively affecting implant success [47].

Despite having results from several studies, we do not yet have a protocol stating the ideal number of implants for full-arch mandibular rehabilitation. In cases of insufficient hard tissue volumes in the posterior sectors, the All-on-4 technique is described as a potential prosthetic rehabilitative solution. Despite the original indications, based on the results of this analysis, this technique may have further indications for use. In fact, its use could, in any case, prevent the problems associated with placing an implant distal to the mental foramen, especially in cases where the mandibular flexion mechanism is increased, such as in brachyfacial patients, with an increased mandibular length, reduced gonial angle, and decreased symphysis bone density, length, and surface area. 

The outcomes of this study and the analysis of different implant rehabilitation models in mandibular full-arch cases show statistically significant differences that result in very interesting clinical data that should be taken into account when planning a patient’s rehabilitation. However, further clinical investigations, that go beyond the retrospective nature of the present study, are needed, using the aid of biometric parameters and with the standardization of implant parameters, such as shape or surface morphology and the prosthodontic superstructure. With this perspective, stronger data may be available showing the effect that this and other factors may have on long-term implant success and stability.

## 5. Conclusions

Based on our study results, we conclude the following:

– In mandibular implant rehabilitations, there is greater susceptibility to bone resorption in implants placed distal to the mental foramen than in mesial ones;
– Key factors in long-term implant success may therefore include the number of implants placed and their location;
– The choice of an “all-on-four” implant placement protocol may be a clinical indication for fixed full-arch mandibular rehabilitations, especially in cases where there is an increased susceptibility to the phenomenon of mandibular flexion.

The results of this study show that it is necessary to investigate this issue further, and further prospective clinical and radiographic analysis should be conducted to confirm these results and investigate the possible underlying causes or concomitant causes of these phenomena.

## Figures and Tables

**Table 1:** Frequencies and percentage of implant position sites.

Implant Position Site	*N*	Percentage of Total
Second Molar	40	4.5%
First Molar	226	25.5%
Second Premolar	84	9.5%
First Premolar	226	25.5%
Canine	66	7.5%
Lateral Incisor	244	27.5%

**Table 2:** Results of χ^2^ test between implant placement models and peri-implant bone loss.

	Bone Loss (*N*, %)		
Model	Yes	No	*p*-Value
1	0 (0.0%)	42 (27.1%)	<0.001
2	38 (24.5%)	29 (18.7%)	
3	13 (8.4%)	13 (8.4%)	
4	8 (5.2%)	5 (3.2%)	
5	5 (3.2%)	2 (1.3%)	

**Table 3:** Results of χ^2^ test between number of implants and peri-implant bone loss.

	Bone Loss (*N*, %)		
No. of Implants	Yes	No	*p*-Value
4	0 (0.0%)	42 (27.1%)	<0.001
6	51 (32.9%)	42 (27.1%)	
8	13 (8.4%)	7 (4.5%)	

**Table 4:** Results of χ^2^ test between implant position and peri-implant bone loss.

	Bone Loss (*N*, %)		
Implant Position	Yes	No	*p*-Value
3.2	0 (0.0%)	122 (13.8%)	<0.001
3.3	0 (0.0%)	33 (3.7%)	
3.4	0 (0.0%)	113 (12.8%)	
3.5	0 (0.0%)	42 (4.7%)	
3.6	25 (2.8%)	88 (9.9)	
3.7	7 (1.5%)	13 (0.8%)	
4.2	0 (0.0%)	122 (13.8%)	
4.3	0 (0.0%)	33 (3.7%)	
4.4	0 (0.0%)	113 (12.8%)	
4.5	0 (0.0%)	42 (4.7%)	
4.6	26 (2.9%)	87 (9.8%)	
4.7	6 (0.7%)	14 (1.6%)	

## Data Availability

All data generated or analyzed during this study are included in this article.
